# Perceived relative social status and cognitive load influence acceptance of unfair offers in the Ultimatum Game

**DOI:** 10.1371/journal.pone.0227717

**Published:** 2020-01-09

**Authors:** Alison Harris, Aleena Young, Livia Hughson, Danielle Green, Stacey N. Doan, Eric Hughson, Catherine L. Reed

**Affiliations:** 1 Department of Psychological Science, Claremont McKenna College, Claremont, California, United States of America; 2 Division of Behavioral & Social Sciences, Claremont Graduate University, Claremont, California, United States of America; 3 The Webb Schools, Claremont, California, United States of America; 4 Robert Day School of Economics & Finance, Claremont McKenna College, Claremont, California, United States of America; Ben-Gurion University of the Negev, ISRAEL

## Abstract

Participants in the Ultimatum Game will often reject unfair resource allocations at personal cost, reflecting a trade-off between financial gain and maintenance of social standing. Although this rejection behavior is linked to executive control, the exact role of cognitive regulation in relation to status cues is unclear. We propose that the salience of status cues affects how cognitive regulation resolves the conflict between financial gain and social status considerations. Situations that tax executive control by limiting available cognitive resources should increase acceptance rates for unfair offers, particularly when the conflict between economic self-interest and social reputation is high. Here, participants rated their own subjective social status, and then either mentally counted (Load) or ignored (No Load) simultaneously-presented tones while playing two rounds of the Ultimatum Game with an online (sham) “Proposer” of either high or low social status. A logistic regression revealed an interaction of Proposer status with cognitive load. Compared to the No Load group, the Load group showed higher acceptance rates for unfair offers from the high-status Proposer. In contrast, cognitive load did not influence acceptance rates for unfair offers from the low-status Proposer. Additionally, Proposer status interacted with the relative social distance between participant and Proposer. Participants close in social distance to the high-status Proposer were more likely to accept the unfair offer than those farther in social distance, whereas the opposite pattern was observed for offers from the low-status Proposer. Although rejection of unfair offers in the Ultimatum Game has previously been conceptualized as an intuitive response, these results instead suggest it reflects a deliberative strategy, dependent on cognitive resources, to prioritize social standing over short-term financial gain. This study reveals the dynamic interplay of cognitive resources and status concerns within this paradigm, providing new insights into when and why people reject inequitable divisions of resources.

## Introduction

Over the past three decades, laboratory studies of “take-it-or-leave-it” bargaining behavior have repeatedly demonstrated that people are averse to unfair allocations of resources [1–3]. In the standard Ultimatum Game scenario, one player (the Proposer) suggests a split of resources with a second player, the Responder. If the Responder accepts, both parties receive the proposed allocations; if the Responder rejects, both parties receive nothing. Traditional economic theory suggests that all non-zero offers should be accepted out of economic self-interest, but about half of Responders in industrialized societies reject highly unequal proposed allocations (e.g., 20% or less) [4, 5]. Researchers have proposed that this rejection behavior reflects an internal trade-off between financial gain and social status considerations, such as reputation maintenance [6] or enforcement of fairness norms [7]. Although resolving this conflict between economic and social outcomes is thought to require cognitive resources, the exact role of executive control in Ultimatum Game scenarios, and how it interacts with social status considerations, has been debated. Because previous studies have manipulated available cognitive resources or social status considerations separately, it is unclear how these two factors differentially contribute to the decision to accept an unfair offer.

According to the influential dual-process framework, acceptance of unequal offers requires cognitive regulation to override a fast, automatic tendency to punish unfair Proposer behavior. These two types of processes—fast and automatic versus slow and deliberative—therefore compete to respectively favor social reciprocation or monetary gain (for a review, see [8]). Overriding intuitive or emotional responses to perceived unfairness requires cognitive regulation [9], a process often attributed to executive control regions of prefrontal cortex [10]. Consistent with this idea, Responders who maximize personal gain by accepting a majority of unfair offers show greater activity in regions of prefrontal cortex associated with cognitive regulation [11], and, conversely, individuals with greater inhibitory control show higher acceptance rates for unfair offers [12, 13]. Thus, it is commonly assumed that the dominant response to an unfair offer is to reject, in line with the *social heuristics hypothesis* that responses favoring fairness may be faster and more intuitive [8, 14].

Nonetheless, other evidence regarding the role of cognitive regulation in accepting unfair offers has been mixed. Experimental manipulations designed to limit cognitive resources have produced varied effects on acceptance rates for unfair offers [8]. For example, De Neys et al. [12] found a decrease in the acceptance of unfair offers using a standard cognitive load manipulation, consistent with the idea that selfishness requires deliberation, but Cappelletti and colleagues [15] found no effect of cognitive load on either Proposer or Responder behavior. More recently, studies using “ego depletion” paradigms, in which performance of a cognitively demanding task exhausts self-regulatory processes prior to the Ultimatum Game decision, have found various effects including increases [16], decreases [17], or no change [18] in the acceptance rate for unfair offers. These results suggest that other sources of variability, including aspects of experimental design, can strongly influence the link between cognitive resources and prosocial behavior [19].

Instead, recent research indicates that the role of prefrontal cognitive regulation circuits may be more complex than just overriding intuitive preferences for fairness. Studies have implicated dorsolateral regions of prefrontal cortex in self-control across a variety of task demands, including dietary regulation [20, 21], intertemporal choice [22–24], and altruistic choices [25]. These data suggest that cognitive regulation helps to orchestrate goal-consistent responses by up-weighting relevant attributes in the decision process [20–25]. Many of these scenarios require eschewing concrete and immediate rewards in favor of options that are more abstract, long-term, or socially removed [26]. Consistent with this idea, disruption of dorsolateral prefrontal cortex via neurostimulation produces *increased* acceptance rates for unfair offers in the Ultimatum Game, even though perceptions of unfairness appear to be unchanged [27, 28]. Therefore, acceptance of unfair offers in the Ultimatum Game may require cognitive regulation to resolve a conflict between concrete, short-term financial rewards and more abstract social consequences which may play out over a longer timescale.

In particular, some researchers have hypothesized that rejection of unfair offers will evolve out of concerns about social reputation maintenance [29–31], since individuals who develop a reputation for accepting unfair offers may receive reduced allocations of resources in future interactions [6]. Another social aspect of the unfair offer in the Ultimatum Game is the introduction of disadvantageous inequality, in which one individual is left worse off relative to the other. Previous research has shown that individuals are generally averse to receiving lower payoffs than their partners [32], especially when they are endowed with lower starting amounts [33]. Finally, punishment of non-reciprocation can vary with the social distance between actors, as seen in phenomena such as *noblesse oblige*, in which high-status individuals are more tolerant of free-riding behavior by low-status partners [34]. Although such concerns about the interpersonal context are arguably irrelevant to the anonymous, one-shot set-up of the typical Ultimatum Game, participants may nonetheless factor these considerations into their acceptance decisions based on learning from real-world experiences where social contexts matter [29].

Correspondingly, some studies have found that manipulating the social context of the Ultimatum Game can change acceptance rates for unfair offers. However, these effects are complex in nature and depend on the nature of the social interactions being emphasized. When group membership is made salient, individuals may be more likely to tolerate unfair offers from in-group Proposers, reflecting in-group favoritism [35]. On the other hand, settings that prioritize concerns about status within the group can decrease acceptance rates for inequitable offers from in-group Proposers [35–37], leading in the extreme to “hyper-fair” rejections of overly generous offers (i.e., more than 50%) [5]. Nonetheless, these status considerations can change dynamically as a function of the social context, even within an individual: for example, a recent laboratory study which manipulated social status within subjects across the course of the experiment found that the same Responders accepted unfair offers at a higher rate when endowed with low rather than high status [38].

In this study we consider how the trade-off between social standing and economic gain is influenced by cognitive regulation. If cognitive regulation in the Ultimatum Game contributes to the decision to favor social standing at the cost of economic gain, it follows that manipulations of cognitive load that reduce available cognitive resources should *increase* the likelihood of accepting unfair offers, especially when concerns about relative social status are high. However, to date this prediction has received little experimental study, with the majority of Ultimatum Game experiments manipulating social status or cognitive load, but not both. Furthermore, depending on whether interpersonal considerations are driven by in-group favoritism or more general concerns about equitable outcomes, the response to an unfair allocation may differ as a function of the relative status of the Proposer and Responder. For example, if Responders are particularly motivated to avoid disadvantageous inequality when their own status is low [33], they should show lower acceptance rates to unfair offers coming from Proposers of higher status. On the other hand, if fairness considerations are driven by in-group bias, acceptance rates should vary with the relative social distance from the Proposer rather than absolute status. Finally, endorsement of social hierarchy has been reported to increase under cognitive load [39, 40], suggesting that increased acceptance of unfair offers may arise from greater automaticity of deference to high status.

To address these questions, we created a variant of the Ultimatum Game in which we experimentally manipulated cognitive load and the social status of a sham “online” Proposer, as well as assessing individual variability in the participant’s own subjective social status. We constrained social status considerations by providing Responders with detailed information about the sham Proposer’s status, as measured by real-world markers of status including age, ethnicity, annual income, and occupation. At the same time, participants provided self-report assessments of their own demographic information, including a commonly-used measure of subjective social status [41]. Last, participants completed a series of survey measures to assess whether cognitive load directly affected the endorsement of hierarchical values, as reported previously [39, 40].

Using this paradigm, we can explore the relative roles of cognitive resources and social status in decisions to accept unfair offers. If cognitive load reduces the ability to resolve conflicts between reputational or status concerns and monetary outcomes, we would predict *increased* acceptance rates under load when such conflicts are high: i.e., in negotiations with a high-status, but not low-status, Proposer. On the other hand, if acceptance rates are uniformly increased under load, it would suggest that cognitive load merely encourages greater acquiescence regardless of Proposer status. Additionally, we can examine the relationship between Proposer status and the participant’s relative social distance, as measured by the absolute difference in subjective social status scores. If acceptance rates vary as a function of in-group favoritism, we would expect higher acceptance rates when social distance is low, regardless of Proposer status. Conversely, if inequality aversion drives rejection of unfair offers, we would expect an interaction of Proposer status and social distance, as concerns about disadvantageous inequality are greater when participants’ social status is low [33]. Finally, by comparing whether endorsement of hierarchical values increased under cognitive load, we can measure the extent to which any effect of cognitive load on acceptance rates reflects reductions in executive function versus increases in attitudes favoring hierarchy [39, 40].

## Materials and methods

### Participants

Undergraduates and members of the local community (N = 157, 86 females, mean age = 20.9 years) participated for partial course credit or monetary compensation. Seven participants were excluded for displaying suspicion about the online player’s existence or motivations in answer to a post-experiment manipulation check, with an additional three participants excluded for exhibiting atypical behavior including: refusing to accept any monetary compensation; failing to comprehend the instructions for the tone counting task; and, exhibiting extreme prejudice toward the online partner based on a stated dislike of the partner’s indicated profession. Thus, data from 147 participants were included in the final analyses. All experimental procedures were reviewed and approved by the Claremont McKenna College Institutional Review Board, and all participants provided written informed consent prior to participation.

### Measures

#### Subjective social status and social distance

Participants were asked to indicate their subjective social status by circling a number on the ladder representation from the MacArthur Scale of Subjective Social Status [41]. The MacArthur Scale has been shown to correlate with measures of psychological functioning and health outcomes, as well as objective socioeconomic status [42, 43]. We derived a subjective measure of social distance by calculating the absolute value of the difference between the participant’s MacArthur Scale score and the sham Proposer’s MacArthur Scale score, as shown to the participant in the personal profile of the “online player” (Low = 1, High = 9).

#### Hierarchy endorsement

To assess whether cognitive load changed participants’ stated endorsement of hierarchical perspectives, we included three survey measures previously linked to hierarchy endorsement under load [40]: the Short Schwartz Value Survey (SSVS) [44], the Moral Foundations Questionnaire (MFQ) [45], and Social Dominance Orientation (SDO_7_) [46]. The SSVS consists of 10 items representing distinct motivational values [44, 47, 48]. Following previous research [40], we created two separate measures for equality values (SVS-ES) vs. hierarchy values (SVS-HS) by averaging relevant items (SVS-ES: Benevolence and Universalism; SVS-HS: Power and Achievement). The MFQ [45] includes five specific subscales that have been grouped into egalitarian versus hierarchical endorsements. Previous research reported that cognitive load was associated with increases in the Authority-Respect (MFQ-A/R) subscale, which measures hierarchy endorsement [40]. We also included two other subscales with particular theoretical relevance to the Ultimatum Game scenario, as described above: Fairness/Reciprocity (MFQ-F/R) and In-Group/Loyalty (MFQ-I/L). Whereas the former reflects egalitarian endorsement, a higher score on the latter subscale is associated with greater endorsement of hierarchy. The SDO_7_ consists of two subscales, Dominance and Equality, which can be summed to obtain an overall hierarchy endorsement score [46].

#### Executive control

We obtained a measure of participants’ subjective executive control using the Effortful Control portion of the Adult Temperament Questionnaire (ATQ) [49]. Given our manipulation of cognitive load though a tone-counting task, we focused our analysis on the ATQ Effortful Control subscales related to effortful attention and inhibitory control. Although it is a self-report measure, the ATQ-EC has been shown to correlate with standard cognitive measures of conflict processing and working memory [50, 51], and is predictive of attentional impairments in borderline personality disorder [52] and attention-deficit/hyperactivity disorder [53].

### Procedure

In this study, we measured the acceptance rate for unfair offers in the Ultimatum Game while manipulating the cognitive load (Load, No Load) of the participant and the social status of the Proposer (High, Low) in a 2x2 between-groups design. To reduce participants’ expectations regarding the purpose of the experiment, the study was advertised in all recruitment materials as an experiment on multi-tasking. Instructions to participants likewise emphasized that the experiment would be examining divided attention, looking at “how well adults are able to accomplish multiple demanding tasks simultaneously.”

**Fig 1:**
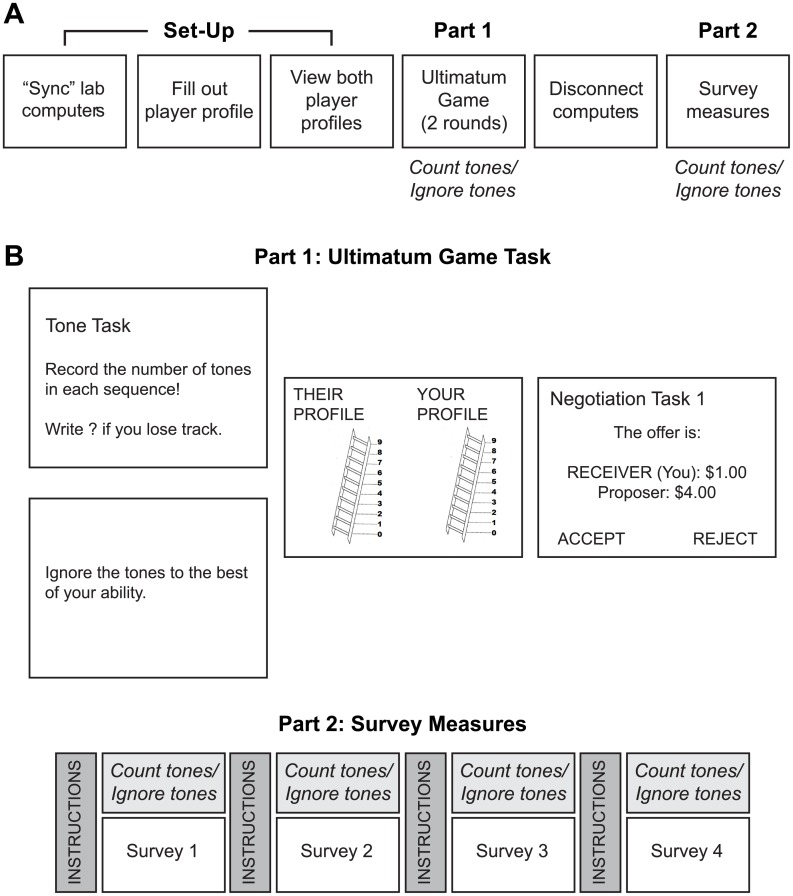
Experimental procedure. A) Timeline of experimental stages. B) Top: Visualization of experimental display in Part 1. After viewing subjective social status information for themselves and an online sham “partner,” Participants played two rounds of the Ultimatum Game while either counting (Load group) or ignoring (No Load group) simultaneously-presented tones. Bottom: Timeline of Part 2, in which participants filled out demographic and survey measures while counting (Load group) or ignoring (No Load group) simultaneously-presented tones.

The experiment consisted of two parts, each of which was completed either under cognitive load or no cognitive load (Fig 1). Participants first played an online Ultimatum Game with a sham “partner” of either high or low status, and then completed a series of questionnaires. To manipulate cognitive load, participants were randomly assigned to one of two groups: a “Load” group in which participants counted differently pitched tones while simultaneously performing the experimental tasks, or a “No Load” group in which participants were instructed to ignore the simultaneously-presented tones. Each experimental session involved single-blind data collection from a single participant, who was randomly assigned to load and partner occupations prior to the start of the experimental session.

The effect of partner status was assessed in two separate experiments run consecutively over a period of two years. In both experiments, the (sham) “online player” was always a middle-aged white male. In the first experiment (n = 76), the “online player” was assigned a high-status occupation (either an orthopedic surgeon or investment banker, counterbalanced across participants) with an annual income over $300,000, placing him in the 98.9^th^ percentile for US household income. In the second experiment (n = 71), the age, gender, and ethnicity of the “online player” were kept constant, but he was given a low-status occupation (either a cashier or fast-food server, counterbalanced across participants) with an annual income under $30,000, placing him below the 25^th^ percentile for US household income.

Across both manipulations of partner status, participants were further randomly subdivided into Load and No Load groups. The Load group (n = 74) performed an auditory tone-counting task, described in detail below, which has previously been shown to tax working memory when performed concurrently with other tasks that rely on shared cognitive resources (e.g., n-back task) [54]. Participants in the Load group were instructed that the tone-counting task should be their first priority. In the No Load group (n = 73), participants heard but did not count the tones. Although participants in the No Load group received the same auditory input, the lack of continuous monitoring should lead to less overlap in working memory demands and, thus, lower interference from the auditory stimuli [54].

The tone-counting task performed by participants in the Load group was modified from [39]. Five audio files of synthesized tones were created: one 20-min file and four 10-min files, each of which had tones sounding every 5 sec. We used four distinct tones that differed only by pitch (700 Hz, 500 Hz, 400 Hz, and 310 Hz). Tones were presented one at a time in sequences of 2 to 7 repetitions, randomly determined, and the order of tone sequences was also randomized. The 20-min file consisted of 44 tone sequences which played continuously throughout the Ultimatum Game and stopped automatically after the participant entered a response to the second offer. Thus, the exact number of tone sequences per participant varied depending on how quickly participants entered their responses to the Ultimatum Game. During Part 2, four separate 10-minute audio files were played, one for each survey, consisting of a total of 100 tones (22–24 tone sequences per file). When the participant completed a survey, the associated audio file would automatically stop playing. Participants mentally counted the number of repetitions in each tone sequence without using fingers or tally marks and they recorded the number on paper at the end of each sequence. Participants were instructed to be as accurate as possible, and that their potential earnings were contingent on accurate performance in the tone-counting task.

In Part 1, participants played two rounds of the Ultimatum Game. They were told that they would “play an on-line negotiation game with a community member located in a laboratory in Los Angeles.” The general structure of the Ultimatum Game was explained, including both Proposer and Responder roles, and participants were required to successfully complete a comprehension check before continuing with the experiment. Participants were informed that they would be deciding on the division of $5.00, but were not provided with any specific examples of how the money would be divided in order to avoid setting specific expectations prior to the start of the experiment. Participants were further reminded that their decisions would affect their monetary outcomes at the end of the experiment.

The Ultimatum Game was presented on the computer using PsychoPy software [55, 56]. To establish a connection with the fictitious “other lab,” the participant waited while the researcher pretended to contact the other lab via cell phone to “make sure the other participant was ready to begin” and viewed a loading screen while the computer “synced” with the other participant’s computer. Once the computers “synced”, a green circle appeared at the bottom right corner of the computer screen; the participant was instructed to inform the researcher if the circle changed to red, indicating a lost connection.

**Fig 2:**
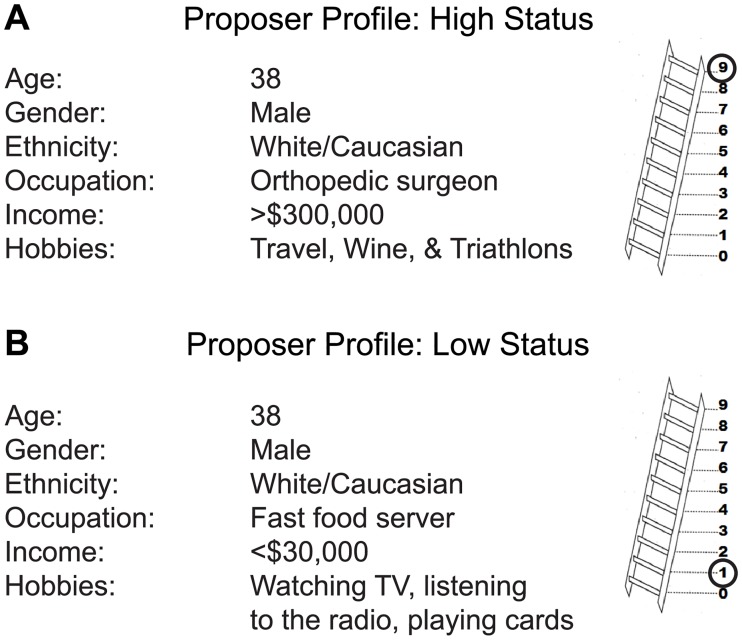
Sample participant information screen for the sham “online partner” of A) high and B) low status. Status of the online partner was established via real-world markers including age, gender, ethnicity, occupation and income, as well as graphical indication by the circled number on the MacArthur scale ladder.

Once the computers “synced”, the participant was prompted to fill out a “player profile” for themselves with personal information to be shared with the online “partner” (Fig 2): age, gender, ethnicity, occupation, annual income, hobbies, and their own social status. The participant was told it was important for both players to know information about each other because they would be accepting or rejecting offers of real money. The participant then received information from the “online player” by viewing both profiles side-by-side on the screen (Fig 2). To maintain credibility, the researcher asked the participant who they were paired with and noted that the Los Angeles laboratory often recruited non-student participants from the community. After confirming that the participant understood the instructions, the experimenter left the testing room.

Next, the participant played two rounds, rigged such that the Proposer role was always assigned to the “online” player and the Responder role was always assigned to the participant. Each offer split $5 of real money. Each participant received two separate offers from the online player: one fair offer (50/50 split) and one unfair offer (80/20 split). Offer order was counterbalanced across participants. The participant received the sum of the two outcomes at the end of the experiment; winnings ranged from $0 to $3.50.

The participant pressed a computer key to begin the Ultimatum Game, at which time the tone file also initiated. Depending on the cognitive load task requirements, the participant was reminded to count or ignore the tones, and then viewed the two player profiles. After the offer appeared, the participant indicated whether he or she accepted or rejected the offer by keypress, and the outcome of the round was presented. Following the second round, the tones stopped and the computer displayed a “disconnecting” screen. The participant informed the researcher that Part 1 was done, and was told how much money he or she had earned from both negotiations.

In Part 2, the participant continued his or her cognitive load task requirements while completing paper-and-pencil survey measures in an attempt to replicate previous findings of increased hierarchy endorsement under cognitive load [40]. Each participant completed three surveys assessing egalitarian vs. hierarchical values, as well as a survey intended to measure executive function (see Measures). Survey order was counterbalanced across participants. All surveys were completed within 10 minutes.

Next, the participant completed a pencil-and-paper manipulation check questionnaire, including questions about household/parental social status and estimated annual income, perceived effort during the experiment, and experience and proficiency with multi-tasking (the stated goal of the experiment). Short answer prompts were used to ask the participant’s opinions about the other player and what he or she thought the purpose of the experiment was. Participants who expressed disbelief or distrust about the player’s existence (e.g., “not real”, “surprised that a man of that age and ‘status’ was participating”) or motivations (e.g., “I thought the guy was pranking me and I would be foolish not to accept any amount of $”) were excluded from further analysis. Although skepticism about the online partner might seem natural, only seven participants explicitly indicated pessimism about the experimental set-up. As a final manipulation check, the participant was asked to recall where they had placed themselves on the ladder representation of the MacArthur scale, as well as the online player’s profile and MacArthur scale status. The experimenter left the room while the participant completed the surveys and manipulation check questionnaire. The entire session lasted approximately 45 minutes.

## Results

### Tone-counting task

As a load manipulation check, we first examined tone-counting accuracy in the Load group. High accuracy on the load-inducing, tone-counting task (mean = 91.5%, SD = 7.14%), confirmed that the dual-task manipulation engaged participants in the Load group. Because tone-counting accuracy was relatively high, we also compared self-reported executive function in the Load and No Load groups using the Effortful Control portion of the Adult Temperament Questionnaire (ATQ-EC)[49]. Participants in the Load group showed a significant decrease in their ATQ-EC scores relative to the No Load group (Load: mean = 0.64 ± 0.11, No Load: mean = 0.69 ± 0.12; t(145) = 2.17, *p* = 0.03). This suggests that the tone-counting task measurably affected participants’ judgment of their cognitive function.

### Ultimatum Game acceptance rates

We addressed whether cognitive load influences acceptance rates for unfair offers in the Ultimatum Game, and to what extent this behavior is modified by the social status of the Proposer vis-à-vis the Responder. As an initial check, we computed the acceptance rates for the fair offer across all load and Proposer status manipulations. Since an even split of money does not engender a conflict between financial and social considerations, we hypothesized that the fair offer would be accepted at a high rate regardless of experimental manipulation. Consistent with this expectation, fair offers were usually accepted across groups facing both low-status (Load = 94.1%; No Load = 91.9%) and high-status (Load = 100%; No Load = 91.7%) Proposers.

Having established that acceptance behavior for fair offers was in line with previous reports, we next examined the roles of cognitive load, Proposer status, and relative social distance in acceptance rates for unfair offers. We conducted a binary logistic regression with unfair offer acceptance (0 = Reject, 1 = Accept) as the dependent variable, and independent variables of cognitive load (0 = No Load, 1 = Load), Proposer status (0 = Low, 1 = High), and social distance. Specifically, social distance was derived from the MacArthur scale and defined as |Status_Participant_ − Status_Proposer_|, where Status_Proposer_ = 1 for the Low status condition and 9 for the High status condition. Data were centered based on the mean social distance across participants (mean = 4.20, SD = 1.63, range = 1 to 7), with far social distance classified as a mean-centered score greater than 0 (n = 70) and close social distance as a mean-centered score less than 0 (n = 77). Gender [57] and offer order [58] have previously been implicated in Ultimatum Game acceptance rates, but preliminary analyses examining their relationship with acceptance rates for unfair offers were not significant (all χ^2^ < 1, *p*s > 0.8). Thus, these covariates were not included in the final model.

Previous work suggests that executive control may be necessary to address the conflict between financial gain and social reciprocation. Thus, cognitive load may be associated with increased acceptance of unfair offers relative to no load, particularly when concerns about social status are high. Additionally, the effects of Proposer status may be reduced or enhanced depending on the relative social distance between the Proposer and Responder. Therefore, we included two interaction terms, Cognitive Load x Proposer Status and Proposer Status x Social Distance. Despite no *a priori* predictions, we also tested a model including the Cognitive Load x Social Distance interaction for completeness, but the additional term did not affect the significance of the other two interactions, and did not appreciably improve model quality, as measured by the corrected Akaike Information Criterion (original model: AICc = 199.1; full model: AICc = 201.3) [59]. Therefore, all reported statistical tests are based on the model with two interactions.

Table 1 displays the results of the binary logistic regression for each stage of the model. From the initial model specification (Block 0) to the final version (Block 2), the model showed an increase in classification accuracy from 59.2% to 63.9%, indicating that the addition of the interaction terms improved prediction accuracy beyond that of models including only the intercept or intercept and main effects (Block 1: 59.9%). Likewise, Block 2 was associated with significant increases in model fit both overall (χ^2^(5) = 12.3, *p* = 0.03) and compared to Block 1 (χ^2^(2) = 9.83, *p* = 0.007), as well as accounting for a greater proportion of variance (Block 1: Nagelkerke *R*^*2*^ = 0.02, Block 2: Nagelkerke *R*^*2*^ = 0.11). These measures suggest that our regression model with interaction terms provides a better fit to the data versus models with fewer parameters.

**Table 1:** Binary logistic regression on acceptance of unfair offers.

Block	Factor	B	SE	Wald	Df	Sig.	Exp(B)
0	**Constant**	**–0.37**	**0.17**	**4.9**	**1**	**0.03**	**0.69**
1	Load (No Load, Load)	0.45	0.34	1.72	1	0.19	1.56
	Proposer Status (Low, High)	–0.24	0.36	0.44	1	0.51	0.79
	Social Distance	–0.09	0.11	0.71	1	0.4	0.91
	Constant	–0.48	0.3	2.56	1	0.11	0.62
2	Load (No Load, Load)	–0.44	0.5	0.78	1	0.38	0.64
	Proposer status (Low, High)	–1.02	0.53	3.67	1	0.06	0.36
	Social Distance	0.23	0.18	1.69	1	0.19	1.26
	**Load × Proposer Status**	**1.59**	**0.71**	**4.96**	**1**	**0.03**	**4.91**
	**Proposer Status x Social Distance**	**–0.54**	**0.24**	**5.08**	**1**	**0.02**	**0.58**
	Constant	–0.24	0.34	0.49	1	0.48	0.79

Bold denotes covariates achieving significance at *p* ≤ 0.05

**Fig 3:**
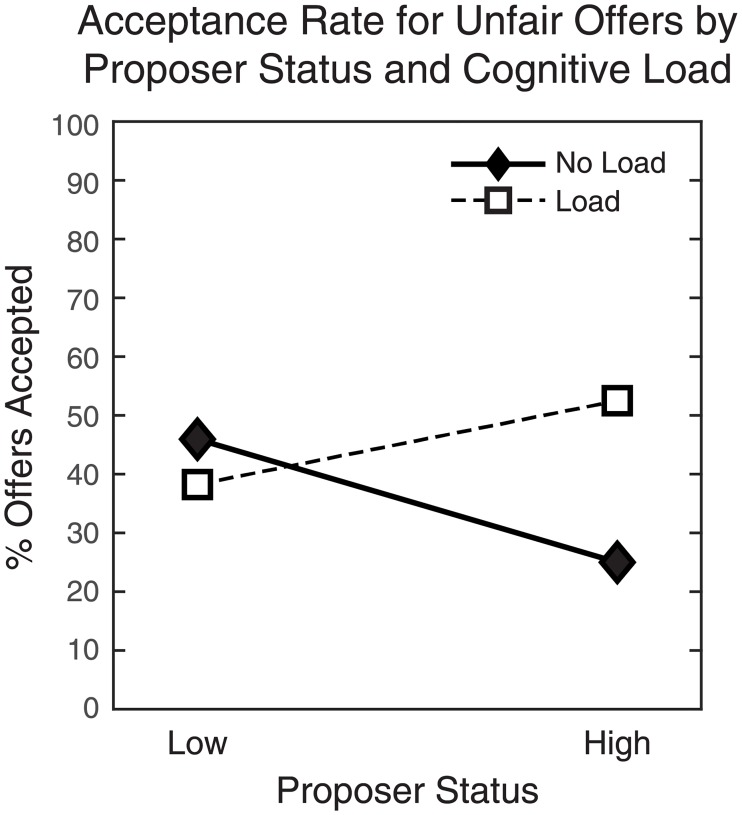
Acceptance rates for unfair offers as a function of Proposer status (Low/High) and cognitive load (Load/No Load).

First, a significant interaction of Load x Proposer Status (*b* = 1.59, Wald = 4.96, *p* = 0.026) was observed. To interpret this effect, we calculated the acceptance rates for unfair offers under Load vs. No Load groups as a function of Proposer status (Low vs. High). Although acceptance rates for unfair offers were generally within the 40–60% range of previous estimates from industrialized societies [5], they varied notably as a function of both load and Proposer status (Fig 3). Whereas acceptance rates for unfair offers from the low-status Proposer were similar regardless of cognitive load (Load: 38.2%; No Load: 45.9%), responses to the unfair offer from the high-status Proposer differed depending on load. Over half of participants (52.5%) who received the unfair offer while performing the tone-counting task chose to accept, yet for the No Load group, the acceptance rate was strikingly lower (25%). Thus, participants in the Load group were twice as likely to accept an unfair offer from a high-status Proposer as their counterparts in the No Load group.

**Fig 4:**
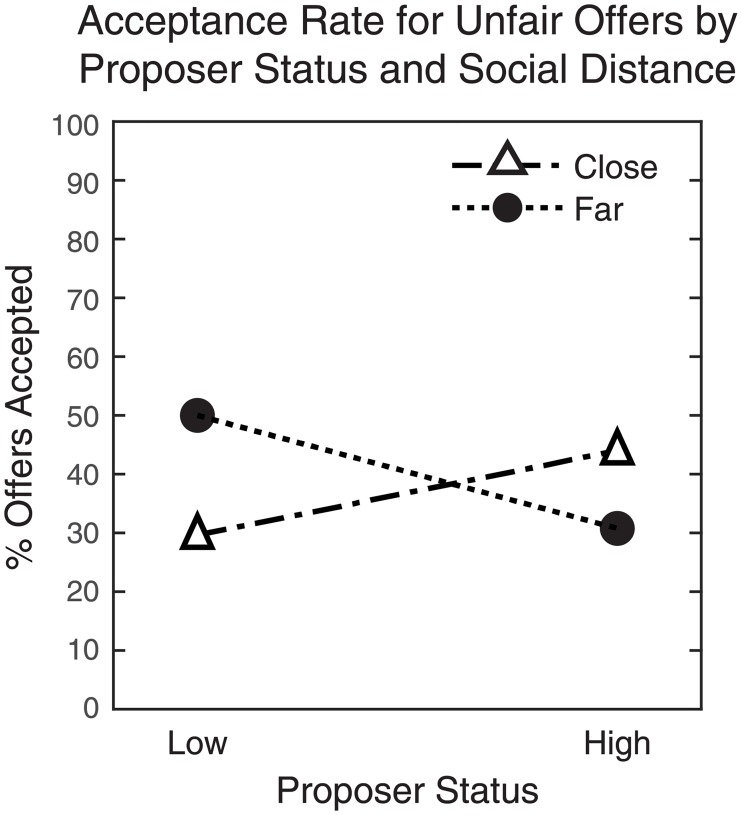
Acceptance rates for the unfair offer by Proposer status (Low/High) and relative social distance (Close/Far).

Second, our binary logistic regression revealed a significant interaction of Proposer Status x Relative Social Distance (*b* = –0.54, Wald = 5.22, *p* = 0.022). High-status Responders interacting with a low-status Proposer (far social distance) were more likely to accept the unfair offer than low-status Responders (close social distance) (Far-Low: 50%, n = 44; Close-Low: 29.6%, n = 27), whereas the opposite pattern was found in the high-status Proposer condition (Far-High: 30.8%, n = 26; Close-High: 44%, n = 50). In other words, Responders who rated themselves lower in subjective social status were less likely to accept unfair offers from both Low (close) and High (distant) Proposers, while Responders of higher status were more likely to accept unfair offers from *both* Proposers (Fig 4).

### Survey analyses

Our results suggest that cognitive load increases acceptance rates for unfair offers, but only from high-status Proposers, in line with heightened conflict between financial gain and concerns about social status in this group. However, another possible mechanism for these results is suggested by previous work reporting that hierarchy endorsement is increased by a variety of manipulations that impair cognitive function, including alcohol consumption, sleep deprivation, ego depletion, and tone counting [40]. Given the increased salience of social hierarchy, these effects would be most pronounced for Responders faced with a high-status Proposer.

We conducted a multivariate analysis of covariance (MANCOVA) with independent factors of cognitive load (No Load, Load) and Proposer status (Low, High), including social distance as a covariate. The dependent variables were scores from three survey measures of hierarchy endorsement (MFQ-A/R, SVS-HS, SDO) and related constructs of fairness (MFQ-F/R), in-group loyalty (MFQ-I/L), and equality values (SVS-ES). No significant effects were found for any factors or covariates, though there was a trend towards significance for cognitive load (F(6,136) = 1.76, *p* = 0.11; Wilks’ ∧ = 0.93, partial η^2^ = 0.07). Inspection of the univariate statistics revealed that this effect was not driven by an increase in hierarchy endorsement, but rather by a significantly *lower* score on the SVS-HS (F(1,141) = 4.23, *p* = 0.04, partial η^2^ = 0.03), from a mean of 5.3 under no load to a mean of 4.79 under load. Therefore, contrary to previous findings [40], we found no evidence in our sample that cognitive load changed acceptance behavior by affecting the level of endorsement for social hierarchy.

## Discussion

Previous research has suggested that cognitive regulation is integral to the decision to punish or accept unfair offers in the Ultimatum Game. However, the literature is divided on whether cognitive regulation contributes to Responder behavior by countering intuitive negative emotional reactions to unfairness [10] or overriding the temptation of economic gain to maintain social standing [27]. Our study provides new insight into this question by experimentally manipulating the availability of cognitive resources, adding information about the social status of the Proposer, and measuring the relative social distance between Proposer and Responder.

First, we found that cognitive load affected the likelihood of accepting unfair offers, but only when Proposer status was high. Paradoxically, this effect was largely driven by a *decrease* in acceptance rates when competing cognitive demands were low. Only 25% of participants under no cognitive load accepted the unfair offer from the high-status Proposer, whereas acceptance rates in the other groups ranged roughly around 40–50%, in line with previous estimates from industrialized societies [5]. Notably, individuals accepted the unfair offer from the low-status Proposer at similar rates regardless of load, ruling out a more general shift in response bias due to limited cognitive resources. Moreover, these results were not explained by an increased endorsement of social hierarchy under cognitive load, in contrast to [40]. Instead, lower acceptance rates for the unfair offer from a high-status partner appear to reflect a deliberate strategy to prioritize social standing over short-term financial gain.

As with many studies of cognitive resource limitation, one question is whether our Load and No Load manipulations differentially affected executive function. In particular, regions of dorsolateral prefrontal cortex associated with cognitive regulation have also been implicated in suppression of sensory responses to distracting, goal-irrelevant items [60–62]. Therefore, it is possible that the No Load condition, in which the tones need to be ignored, may burden executive function to a similar extent as the Load condition, in which tones are counted. While this possibility cannot be ruled out, we feel it is unlikely to account for the present data. Previous research that compared tone-counting in a single- versus dual-task setting has shown that performance decrements associated with multitasking interference depend on the extent to which dual tasks rely on overlapping, as opposed to separate, cognitive resources [54]. Thus, when auditory tone-counting was combined with a visual tracking task, counting performance was unaffected. However, tone-counting performance worsened when combined with a concurrent 2-back paradigm, presumably due to the overlapping demands on working memory resources [54]. Notably, these effects were observed using a tone-counting paradigm in which participants were instructed to only count high pitch tones while ignoring low pitch tones, suggesting that ignoring irrelevant auditory tones need not tax cognitive resources *per se*. Consistent with this idea, research in the domain of selective visual attention suggests that the distracting effect of secondary auditory input is greater when it must be actively encoded and maintained than when it is ignored [63].

Nonetheless, our inferences regarding the effect of the load manipulation would be strengthened by having additional independent measures of self-reported executive function. Although the Load group showed a significant decrease in subjective effortful control on the ATQ-EQ, relative to the No Load group, it should be noted that the ATQ is typically employed to measure stable temperament traits rather than state effects. Therefore, interpretability of this measure would have been bolstered by the inclusion of a baseline ATQ administration prior to the experiment in order to more directly distinguish the effects of load manipulation versus pre-existing group differences. The omission of such a baseline measure is a limitation of the current design. However, we failed to find a significant main effect of load group in any specification of the model, suggesting that variation in executive function between groups cannot fully account for the differential effects of cognitive load on acceptance rates.

Second, we found a significant interaction of Proposer status and relative social distance. Critically, participants with high self-reported subjective social status were more likely to accept the unfair offer regardless of relative social distance from the Proposer, whereas low-status Responders showed the opposite pattern. These results are inconsistent with a general effect of in-group favoritism [35] or enforcement of intragroup reciprocity norms [36]. Rather, low-status Responders may be particularly sensitive to disadvantageous inequality, leading to lower acceptance rates for unfair offers regardless of the source [33]. Likewise, although the high-status Responders appear to demonstrate greater tolerance of in-group non-reciprocation from a high-status Proposer [35], in-group favoritism cannot account for the high acceptance rates for offers from the low-status Proposer. One possible alternative is the idea of *noblesse oblige*, that high-status individuals may be more willing to accept non-reciprocation from individuals of lower social rank [34]. However, it is worth noting that more individuals in our sample self-reported high subjective status, so these results must be interpreted with care. Future research examining the role of social distance in the Ultimatum Game should use larger, more heterogeneous samples with greater variation in age, household income, ethnicity, and education.

Additionally, future studies could exogenously manipulate participant status in the laboratory, for example by endowing one participant with a greater amount of money (e.g., [33]). However, it is important to note that artificial status distinctions can alter expectations about whether reciprocity is appropriate. For example, when Proposers are told that, based on their performance on another task, they have “earned” the right to allocate resources in the Ultimatum Game, they tend to make lower offers without an appreciable decrease in Responder acceptance rates [64]. This shift may explain the difference between our findings and previous Ultimatum Game studies manipulating status in the laboratory [38, 65], which have reported increased acceptance rates for unfair offers when social status is low. Because these studies manipulated status through competitive tasks, participants may have internalized expectations regarding their right to the resources allocated in the Ultimatum Game. In contrast, our experimental design utilized real-world measures of social status, allowing us to tap into participants’ lifelong experience of social context.

More generally, our results suggest that cognitive regulation orchestrates the Responder’s response to unfair allocations by altering the relative weighting of financial and social outcomes. Thus, the social context of the Ultimatum Game may differentially influence whether cooperative or selfish behavior is the “intuitive” response to non-reciprocation [8]. When Proposer status is low, acceptance behavior does not depend on availability of cognitive resources, consistent with the idea that status concerns are less pronounced in this condition. In contrast, a similar acceptance rate from the high-status Proposer is seen only under cognitive load, reflecting the heightened salience of the trade-off between financial gain and social status in this interaction. Similar to previous findings linking disruption of cognitive regulation systems to increased acceptance of unfair offers [27, 28], our results suggest that caution may be warranted when making inferences about acceptance of unfair offers in terms of a deliberative override of automatic rejection behavior [e.g., 10, 12, 13].

Instead, changes to the Ultimatum Game set-up may shift the salience of financial and social outcomes, altering the effects of cognitive demand. For example, when the financial stakes at play are raised, increasing the temptation of short-term economic gain, Responders generally show higher acceptance rates for unfair offers [66–68]. In this case, as in the low-status Proposer condition, we may expect that participants would show less effect of cognitive load. On the other hand, heightened conflict between financial reward and social standing could potentially place a ceiling on selfish behavior, explaining persistent observations of fairness considerations even at extremely high (though hypothetical) stakes [69]. Concerns about disadvantageous inequality might cause low-status Responders to reject even relatively large resource allocations from high-status Proposers if they reflect a smaller share of the pie (cf. “hyper-fairness”, [5, 30]). Further research should investigate the interaction of social distance and financial stakes in Ultimatum Game acceptance behavior, and how these effects are moderated by cognitive demand.

The robustness of rejection behavior (i.e., lower acceptance rates for unfair offers) in the anonymous one-shot Ultimatum Game is often cited as an example of the power and pervasiveness of inequality aversion [4, 70]. However, our data suggest that paradigms which withhold information about the other players may affect participants’ strategic considerations, increasing uncertainty about the trade-off between reaping economic gains and asserting social status. Although weighing social status concerns does not always require executive control, as seen in the low-status Proposer condition, the extent to which executive control is needed may vary depending on the salience of social status. In the case of the anonymous one-shot Ultimatum Game, participants are usually instructed that they are playing other people like themselves. In this case, the salience of social status becomes an endogenous variable, highlighting the role of people’s perceptions of their own relative social status in acceptance behavior for unfair offers. Therefore, our data potentially provide a clue as to why there is such variability in the effects of status and cognitive regulation across the literature [8]. Future research should further examine how individual perceptions of social status affect Ultimatum Game decisions, perhaps in combination with metrics of physiological arousal and attention (e.g., eye-tracking).

In conclusion, we examined the influence of cognitive load, Proposer social status, and relative social distance on Responder behavior in the Ultimatum Game. Limiting cognitive resources through load increased the likelihood of accepting unfair offers from a high-status Proposer relative to no load, consistent with the idea that cognitive regulation is necessary to resolve the conflict between social status concerns and economic self-interest. In contrast, no effect of load was observed in interactions with a low-status Proposer. At the same time, we observed an interaction between Proposer status and relative social distance, with individuals high in self-reported status being more likely to accept the offer from both low- and high-status Proposers, and low-status individuals showing the opposite effect. Further research manipulating Proposer and Responder status along with cognitive demands will shed further light on when and why unfair offers are accepted.

## Supporting information

S1 File File containing experimental data set. (CSV)
